# Empowerment of individuals in Iranian health systems: a qualitative study using the Z-cognitive map approach

**DOI:** 10.1186/s12913-024-10866-8

**Published:** 2024-04-03

**Authors:** Mostafa Izadi, Hamidreza Seiti

**Affiliations:** 1 National Center for Health Insurance Research, Tehran, Iran; 2 Department of Industrial Engineering, Iran University of Science and Technology, Tehran, Iran

**Keywords:** Insured empowerment, Health system, Cognitive map, Z-number, Content analysis

## Abstract

The empowerment of people is considered as one of the most effective approaches in national healthcare systems. Identifying the effective criteria for this empowerment approach can be useful for planning enhancements. Therefore, studying and researching different aspects of people empowerment, and identifying the various relationships among related factors are of great importance. In this study – after identifying and extracting the effective factors in empowering individuals/insured persons, and interviewing health insurance and healthcare experts through content analysis – a causal model examining variables and their impact intensity through cognitive mapping is designed and drawn up. In modeling the concept of empowerment, to cover the ambiguity of expert comments, a combination of the Z-number approach with cognitive mapping has been used. Results demonstrate how various factors relate to insured empowerment. According to the results of empowerment strategies, the insurance participation strategy with the highest central index was determined as the most effective strategy, and the appropriate component for individuals gained the highest score in the centrality index. The results of this article help a lot to policy making in medical insurance.

**Supplementary Information:**

The online version contains supplementary material available at 10.1186/s12913-024-10866-8.

## Background

Today, there are many barriers to achieving universal healthcare in society: resource constraints, health service discontinuities, and lack of focus on the individual. Additionally, factors such as the changing population age pyramid, increasing life expectancy, changing treatment patterns, increasing treatment costs, prolonged lifespans, poor quality treatment, medical errors, restrictions on access to healthcare, and economic fluctuations can all exacerbate dissatisfaction. The WHO's response to such a situation is to design an overall strategy for person-centered health services that focuses on the health needs and expectations of individuals and communities, rather than focusing on disease or treatment. This approach extends the concept of the patient-centered approach to individuals, families, associations, and society and emphasizes the health of community members and their key role in shaping policies and services [1]. The use of the empowerment approach has been considered one of the most effective measures in promoting health indicators and enhancing quality of life. The WHO accepted empowerment in 1986 as a critical initiative to address global health inequality [2, 3]. Empowerment can also be described as a process of removing power imbalances between people [4]. To date, numerous approaches and strategies have been adopted to empower individuals within communities, which can be categorized into six general strategies: (1) information and education; (2) accountability; (3) financial empowerment; (4) advocacy; (5) inclusion and participation; and (6) local organizational capacity [5]. These approaches have been applied in more than 100 countries, leading to many improvements in empowering people. First, the purpose of information and education strategies is multifold: to equip people to make better use of opportunities; to develop better access to health services; to secure and safely exercise a range of human rights; to make wise decisions and actions; and to promote overall health literacy. This strategy depends on various factors, such as education, personal abilities, working life, culture, language, and gender [6, 7]. Second, accountability means being able to answer questions from individuals and organizations with regard to decisions and actions taken from a nonmedical perspective [8]. This approach allows individuals to respond to and solicit the behaviors of service providers, policymakers, and health activists. Third, the World Health Report [9] contains documents that focus specifically on health system financing and empowerment. This approach uses financial mechanisms to increase the capital of families and communities or to help manage their spending. Subjective health service subsidies, income generation schemes, condition-based cash exchanges, and microcredit are some of these mechanisms. Fourth, the purpose of advocacy is to empower individuals from both a legal and informational perspective and to increase the power of individuals and groups to make service providers more accountable than other outcomes. This approach endeavors to expand the range of individuals' choices by providing necessary information and solutions and increasing their decision-making power [10]. Fifth, the purpose of the partnership approach is to encourage participation and improve the level of involvement of all health system actors in providing fair treatment, as well as extending it to marginalized groups [11]. That is, the participation of people is a result of the operationalization of other strategies. Sixth, the organizational and local capacity building approach is managed and resolved through the mobilization of organizations, collective actions, group meetings, and the rotation of medical resources to address the health issues of individuals and society [12]. Considering the importance of empowering people, it is important to plan and develop policies. To plan and make policy in this field, it is important to identify and determine the variables that affect individuals' empowerment in each of the strategies mentioned. The purpose of this study was to identify the causal relationships between each of the strategies and variables affecting individuals' empowerment in the healthcare system. The results can then be used by policymakers in these areas. The structure of this article is as follows: The following section reviews the relevant literature, focusing on empowerment in the field of health and the healthcare system. Next, by focusing on expert knowledge with the Z-number cognitive map (ZCM) approach, we seek to identify the causal and affective relationships affecting the empowerment of insured persons/health insurers in Iran. The paper concludes with a discussion, conclusion, and suggestions for future studies. The present study uses cognitive mapping to model factors affecting individual empowerment within the health system.

Research into empowerment is extensive and employs various theoretical and practical approaches. However, even with this diversity, this literature suffers from various limitations. Most importantly, there is no agreement on the definition of empowerment, and without an integrated understanding, empowerment will remain a concept that will be difficult to use [13]. Empowerment studies in various disciplines are based on the proposition that, to improve quality of life, both at work and at home, individuals must be able to enact changes. People need to make these changes not only in their personal behavior but also in the social environment around them, as well as within situations and organizations that affect their lives. There is extensive interdisciplinary research literature on the concept of empowerment that reflects its multiple meanings. Concepts of empowerment have been used in business management and economics [14], international development [15], psychology [16], sociology [17], medicine [18], nursing [19], social work [20] and rehabilitation [21]. Empowerment is a dynamic, positive, interactive, and social process that is shaped by others and will lead to better quality of life. For this reason, in recent years, this concept has become a necessity in healthcare system research [22]. As applied in healthcare fields, empowerment is used in a number of different ways. It has been termed the backbone of health promotion, as well as providing methods for increasing patient autonomy and participation in decisions. With the rise of chronic diseases, empowerment has been strategically implemented to encourage patient participation and healthcare responsibility, as well as to improve health outcomes and to lower costs [23]. Empowerment is a communication structure (for example, in physician‒patient counseling) associated with concepts of power, rights, justice, and situational control, thus demonstrating the capacity to solve problems and obtain a fair share of resources [24, 25]. Health empowerment is the process by which an individual identifies his/her problems, makes informed decisions about health management, considers reasonable goals and appropriate ways to achieve those goals, and tackles the related challenges [26, 27]. To date, various definitions of empowerment have been proposed in health systems. Empowerment, as a concept, views patients as self-determining agents, with at least limited control over their health [28]. It involves a process and outcome, with communication among patients and healthcare professionals involving illness-related information resource sharing, thus increasing patient feelings of control, self-efficacy, coping mechanisms, and the ability to influence their condition(s) [3]. Building motivation and capacity (skills and knowledge) is a key part of empowerment, allowing patients to be part of decision-making processes, consequently rebalancing power dynamics between patients and health workers [29]. It is also designed to assist the growth of self-directed behaviors, for example, by encouraging and supporting self-reflection on their experience of different health conditions. This construct promotes multiple beneficial characteristics, such as psychological safety, collaboration, warmth, and respect, which are crucial in laying the groundwork for self-directed positive behavioral and emotional change [30]. Patients who are empowered can communicate and instigate reform, regulate self-care, develop requisite coping strategies, skills and potentials, and self-manage independently, thus demonstrating control over their own lives [31]. The WHO [32] description of empowerment states that it is "a means through which people obtain greater control over actions and decisions influencing their health, and as such individuals and communities demand to develop abilities, have access to information and resources, and the chance to participate in and influence the factors that affect their health and well-being”. To date, many studies have been performed that have investigated the effect of empowerment on different groups of patients. Köhler et al. [33] examined the impact of empowerment on cardiovascular patients. Madmoli [34], and Gómez et al. [35], examined the effect of empowerment on diabetic patients. John et al. [36], evaluated the impact of empowerment on chronic disease management. Tabari et al. [37], examined the effect of empowerment on elderly individuals with chronic obstructive pulmonary disease. Bravo et al. [38], presented a theory of patient empowerment, which includes a systematic approach, including access to health care as an element of the empowerment process. In this model, each level of empowerment is seen as a contributing factor to patient empowerment. The individual level of the patient reinforces the fact that the patient is entitled and must accept responsibility for his or her health. Healthcare professionals should create an environment that supports patient empowerment by building a shared relationship of shared decisions that helps respect patient rights. The ability to create this collaborative environment relies on a responsive health care system that has access to care (including chronic disease self-management programming, personal care planning, and patient education). A systematic approach, with the help of various empowerment indicators, thus empowers the patient and plays an important role in the individual empowerment process. Patient empowerment has clinical implications, such as the ability to adapt to chronic illness, quality of life, independence, and health status.

## Methods

**Fig. 1:**
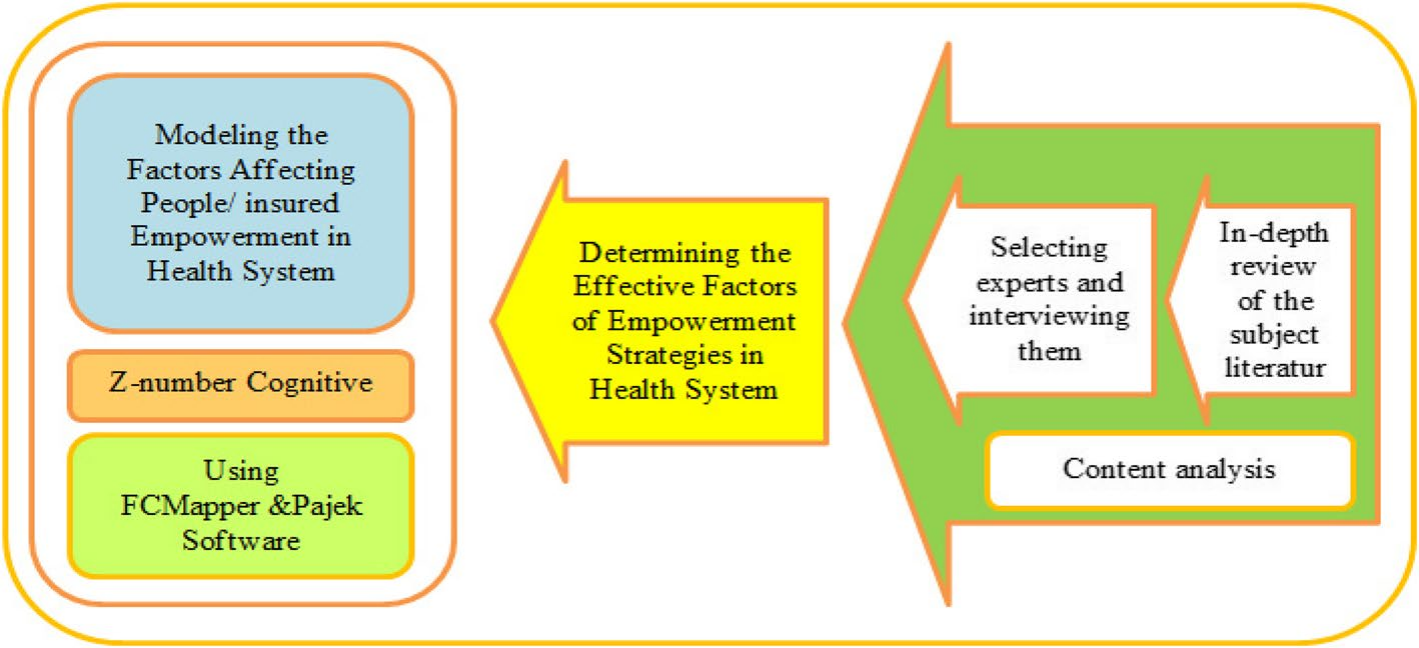
Research road map

The approach used in this article is the use of the combined method of fuzzy mapping and the Z-number method, which has been used in the study of foresight [39]. In this study, qualitative data were derived from implicit expert knowledge. After analyzing the relevant literature and interviewing experts, the factors affecting empowering individuals were identified and extracted using the content analysis method. After identifying and extracting the variables, they were merged and refined. The interview with the experts included 30 open-ended questions based on the Likert scale and the main strategies of empowering people in the health system and was designed by the researcher and covered the components affecting each of them. The questionnaire used in the interviews with experts is included in the section of Supplementary file 1. The interview was tested on a trial basis with three experts and changes were made based on that and then its validity was approved by the group of experts. Reliability is a crucial aspect in qualitative research, and the literature review highlights several methods to achieve it [40]. These include providing accurate guidance during the interview process for effective data collection, establishing structured processes for conducting and interpreting convergent interviews, and involving specialized committees. In this research, these considerations were taken into account during the interviews to enhance reliability. Given that this research falls within the field of inference in soft operations, the reliability of the findings was assessed using retesting and calculating the correlation between the data. It is important to note that the validity and confirmation of the model used in this research are directly associated with the quality of conducting interviews and extracting indirect data from experts. Paying attention to the aforementioned methods can effectively contribute to enhancing the validity of the findings. Following the completion of the study, the obtained results were reviewed and approved by the participating experts. Subsequently, the results were presented to the Iran Health Insurance Organization, which functions as the largest medical insurance organization in the country. An interview was conducted with 12 experts who had PhD degrees and had more than 10 years of executive experience in Iran's health insurance organization and lasted about 30 min. In this paper, the Z-Number hybrid approach in fuzzy perceptual mapping is then used to help the expert understand the problem and to eliminate uncertainty and ambiguity in the experts' comments about the variables and their cause-and-effect relationships. Figure 1 illustrates the steps of the study.

### Z-Number Cognitive Map (ZCM)

The basis of the proposed model in this study is to extract implicit data from experts based on content analysis. This model is applicable when the number of problem variables studied is relatively high, meaning that experts are unable to directly identify how the variables are related. The model developed by this paper is based on the theory of fuzzy cognitive automatic mapping (based on graph theory) and data generated by experts (first presented by Rodriguez-Repiso et al. [41]). In the proposed method, ambiguity and uncertainty were considered by experts. Using the Z-number approach and its combination with the fuzzy cognitive mapping model, the paper attempts to enhance the degree of reliability. The findings of the proposed model are as follows:

- Step1. Selecting subject-related experts

It is first necessary to select relevant experts. The quantity of experts should be implicitly related to the complexity level of the problem being studied. To cover the dimensions of the issue fully, there should be a minimum of five experts involved. Each comment by a selected expert may have a different level of importance. The ultimate decision-maker is responsible for defining each expert’s significance. Each expert’s weight of importance should then be normalized after determining their individual importance for calculation purposes.

- Step2. Identifying effective subject variables

This stage makes use of various techniques, including brainstorming and Delphi, to expand certain subject dimensions. Related variables are then identified. As the problem involves high levels of ambiguity, there may be a relatively high number of variables. Those that occur with higher frequency among expert comments shall be chosen as effective variables.

- Step3. Initial matrix creation

O, the initial matrix, is an [N × M] matrix, within which N shows the number of key factors (referred to as concepts or variables), and M represents the quantity of interviewees. The O_ij_ element represents the level of importance that an expert (j) assigns to concept (i) in comparison with different concepts/variables. To address the ambiguity in expert commentary, importance is formulated in terms of verbal statements using a Z-numbers approach. Each element O_i1_, O_i2_…, O_im_, is verbally stated as a vector Vi element associated with concepts/variables belonging to matrix row (i).

- Step4. Determining variable importance

Next, the importance of each individual variable shall be determined. Because experts do not affect one another's opinions, the degree of importance is independently determined by each expert using a Z-numbers approach. The final degree of significance of each variable is thus obtained by computing each expert’s weight via the weighted sum method.

- Step5. Obtaining a definite matrix

The initial matrix is then converted to a definitive matrix. The derived importance factor of variables, as well as the expert importance factor, must be taken into account. The final definite matrix is then constructed from the matrix elements. Within this matrix, the numbers range from zero to one.

- Step5.1. Creating a strength of relationships matrix (SRM)

The strength of the relationship matrix may be stated as a matrix with dimensions [N × N]. Rows and columns relate to key matrix factors. Each matrix element demonstrates a relationship between factor (i) and factor (j). ${{\text{S}}}_{{\text{ij}}}$ values within a range [-1, 1] can be accepted. Key factors are shown as numerical vectors in the form of ${{\text{S}}}_{{\text{i}}}$, with N elements per variable on the map. Three possible relationships exist between concepts (i) and (j):

- ${{\text{S}}}_{{\text{ij}}}>0$ demonstrates a direct (positive) cause‒effect relationship between (i) and (j). Thus, increasing concept (i) value increases concept (j) value.
- ${{\text{S}}}_{{\text{ij}}}>0$ demonstrates a reverse (negative) cause‒effect relationship between (i) and (j). Thus, decreasing concept (i) value increases concept (j) value.
- ${{\text{S}}}_{{\text{ij}}}>0$ demonstrates no relationship between (i) and (j). Three parameters must therefore be considered when determining values. ${{\text{S}}}_{{\text{ij}}}$ shows the (i) and (j) relationship. The power of  ${{\text{S}}}_{{\text{ij}}}$ indicates how powerfully concept (i) can affect concept (j), as well as the causality direction, which shows whether concept (i) influences (j) or vice versa.

- Step5.2. Determining the Duality of Relationships

Vectors ${{\text{V}}}_{1}$ and ${{\text{V}}}_{2}$ are associated with factors 1 and 2; thus, ${{\text{X}}}_{1}$(${{\text{V}}}_{{\text{j}}}$) and ${{\text{X}}}_{2}$(${{\text{V}}}_{{\text{j}}}$) signify the membership degrees of ${\text{j}}$ within vectors ${{\text{V}}}_{1}$ and ${{\text{V}}}_{2}$. Hence, these vectors display a positive correlation (a direct link between 1 and 2, with ${{\text{S}}}_{{\text{ij}}}>0$). If, for the entirety or the majority of elements associated with these two vectors, ${{\text{X}}}_{1}$(${{\text{V}}}_{{\text{j}}}$) remains similar to ${{\text{X}}}_{2}$(${{\text{V}}}_{{\text{j}}}$), while vectors ${{\text{V}}}_{1}$ and ${{\text{V}}}_{2}$ maintain an exclusively negative relationship between 1 and 2, and if for the entirety or the majority of elements associated with the two vectors ${{\text{X}}}_{1}$(${{\text{V}}}_{{\text{j}}}$) are similar to (${{\text{X}}}_{2}$(${{\text{V}}}_{{\text{j}}}$) -1), then ${{\text{S}}}_{{\text{ij}}}<0$.

- Step5.3. Determining the strength of relationships matrix (SRM)

Concerning the similarity between vectors V1 and V2, a proximate relationship between these two vectors confirms the relationship between concepts 1 and 2 and is thus signified by ${{\text{S}}}_{12}$. The relationship proximity of the two vectors is based on the inter-vector distance. Various calculations are necessary for positively and inversely related vectors. If ${{\text{V}}}_{1}$ and ${{\text{V}}}_{2}$ are positively correlated, then the closest distance j (j = 1…, m) occurs when ${{\text{X}}}_{1}$(${{\text{V}}}_{{\text{j}}}$) = ${{\text{X}}}_{2}$(${{\text{V}}}_{{\text{j}}}$). If dj represents the distance between elements of j, the vectors ${{\text{V}}}_{1}$ and ${{\text{V}}}_{2}$ are stated as follows:

1 $${{\text{d}}}_{{\text{j}}}=\left|{{\text{X}}}_{1}\left({{\text{v}}}_{{\text{j}}}\right)-{{\text{X}}}_{2}\left({{\text{v}}}_{{\text{j}}}\right)\right|$$

AD then indicates the mean distance between ${{\text{V}}}_{1}$ and ${{\text{V}}}_{2}{\text{vectors}}$:

2 $${\text{AD}}=\frac{\sum_{{\text{j}}=1}^{{\text{m}}}\left|{{\text{d}}}_{{\text{j}}}\right|}{{\text{m}}}$$

S, or the proximity/similarity between two vectors, is represented with this equation:

3 $${\text{S}}=1-{\text{AD}}$$

$\mathrm{S }=1$ confirms total similarity, while $\mathrm{S }=0$ shows maximum non-similarity.

If the ${{\text{V}}}_{1}$ and ${{\text{V}}}_{2}$ vectors are inversely correlated, then the method used to calculate similarity is the same as previously used, with an exception being that, in this case, the equation for calculating the distance between relevant elements is inversely related to vectors ${{\text{V}}}_{1}$ and ${{\text{V}}}_{2}$  .

4 $${{\text{d}}}_{{\text{j}}}=\left|{{\text{X}}}_{1}\left({{\text{v}}}_{{\text{j}}}\right)-(1-{{\text{X}}}_{2}\left({{\text{v}}}_{{\text{j}}}\right)\right|$$

All remaining equations similarly calculate the mean distance between two vectors (AD) using Eq. (8) and their similarity (S) using Eq. (9).

In this case, ${\text{S}}=1$ demonstrates complete inverse similarity between two vectors, while ${\text{S}}= 0$ indicates noncomplete inverse similarity. During the study, in terms of relations between numeric vectors, neither complete similarity nor complete non-similarity is predicted. For each ${{\text{V}}}_{1}$ and ${{\text{V}}}_{2}$ pair of vectors, $-$ the similarity between the two vectors is calculated twice, once based on a positive relationship, and next based on an inverse relationship. Higher similarity confirms a dual relationship between the crucial factors of success (i) and the key factors (j) of a positive/direct or negative/inverted relationship. The strength of the relationship in defining the ${\pm {\text{S}}}_{{\text{ij}}}$ sij value is introduced in the SRM.

- Step6. Creating the final communication matrix

After the SRM matrix is complete, some data contained therein may be misleading. Not all key factors in the matrix are interrelated, nor is a causal relationship always present. Experts must further analyze the data, converting the SRM matrix to a final matrix, which contains only fuzzy numerical elements signifying causal relationships between critical factors. In SRM matrix data analysis, two vectors can be mutually related. Vectors could indicate close relationships mathematically but be completely unrelated logically. Such atypical relationships would be identified easily by expert opinion.

- Step7. Fuzzy cognitive map graphical representation

The graphical representation of the ultimate success matrix, similar to an FCM, gives a useful FCM that indicates crucial success factors. In the final display, each arrow related to factors (i) and (j) possesses a marked weight. This value demonstrates the power of the direct/inverse causal relationship among elements, as well as the final value contained in the matrix of success. This figure is presented in the cell marked by row (i) and column (j).

## Results

In this section, using the Z-number cognitive mapping method introduced in the previous section, cause and effect relationships are identified among the various factors affecting the empowerment of individuals/insured in the healthcare system.

**Fig. 2:**
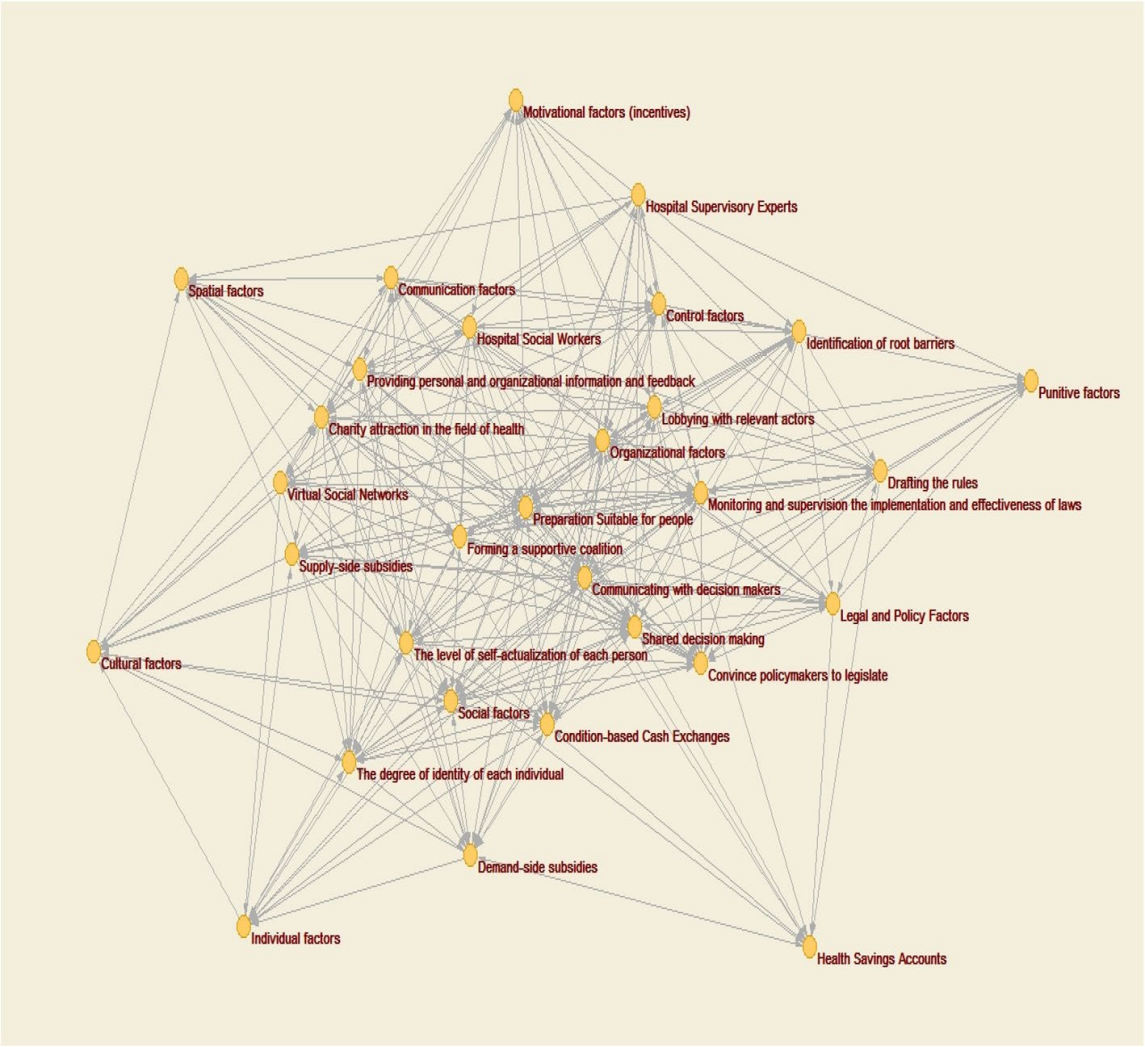
The cognitive map of the health insurance empowerment model

As the first step of the proposed method, experts on the topic of empowering people in the health system should be selected. The number of experts selected is directly related to the complexity of the problem under study. The number of experts used in this study is 12, all of whom are affiliated with university education, are recognized as experts, and have a history of working with the Iran Health Insurance Organization. According to the second step of the proposed method, different techniques, such as brainstorming and Delphi, are used to determine different aspects of empowering people in the health system (based on the six strategies presented). As a result, the variables associated with each of these strategies are identified (see Table 1). Since the problem is in a highly ambiguous space, the number of variables can be relatively large. The variables that are most abundant among experts are selected as effective variables. Since the number of empowerment variables is 30 and the experts used are 12, the primary matrix on empowerment measures [30 × 12]. According to step 4, the importance of each variable is determined by the use of verbal variables, and – based on the Z-number approach – this is carried out independently by each expert. According to step 5, a strength of relationships matrix is drawn up, measuring [3 × 30]. Rows and columns are related to the matrix of effective factors in empowering people in the healthcare system (as shown in Table 2). According to step 6, the final communication matrix is then obtained (see Table 3). The cause-and-effect model of insured empowerment is drawn by Pajek ver. 5.08 software and is shown in Fig. 2. The calculations are then performed using FCMapper_bugfix_27.1.2016 software, and the results are presented in Table 4. The components investigated in empowerment have a direct and positive relationship with each other. The components with the highest centrality index score in each strategy are now examined. In the information and education strategy (with the aim of informing the insured), the component with the highest score of 22.09 (social factors) is thus given the highest centrality index. In the strategy of accountability of insured service providers, the component with a 22.77 score (organizational factors) has the highest centrality index. Organizational factors here are a set of factors that can be applied by the insurer as a service buyer to make all service providers more accountable. In the strategy of financial protection of the insured, the component with the highest score of 17.17 (condition-based cash exchange) has the highest centrality index. This component includes all monetary incentives that can be used by governments to encourage people to participate more and better in community health programs (for example, donations of money for the regular vaccination of children, participation in training programs for pregnant mothers, etc.). In the strategy of protection of insured rights, the component with the highest score of 17.05 (hospital social workers) has the highest centrality index. Hospital social workers, with an understanding of the relative rules of hospital and health insurance, can play an important role in informing patients and can also play a role as patient advocates when needed. The components with the highest and lowest centrality scores in each of the strategies are listed in Table 5. As stated in the insured participation strategy, the components with a score of 32.01 and score of 23.05 (appropriate support for individuals and co-decision making, respectively) have the highest scores in the centrality index. These two components indicate the result of health system empowerment.

**Table 1:** Strategies and variables related to insured empowerment

Number	Strategy	Number	Component
1	Information and education (Informing the insured)	1	Individual factors
		2	Communication factors
		3	Spatial factors
		4	Cultural factors
		5	Social factors
2	Accountability of insured service providers	1	Legal and policy factors
		2	Punitive factors
		3	Control factors
		4	Motivational factors (incentives)
		5	Organizational factors
3	Financial protection of the insured	1	Health savings accounts
		2	Condition-based cash exchanges
		3	Supply-side subsidies
		4	Demand-side subsidies
		5	Charity attraction in the field of health
4	Protection of insured rights (advocacy)	1	Drafting the rules
		2	Hospital social workers
		3	Hospital supervisory experts
		4	Virtual social networks
		5	Convince policymakers to legislate
5	Insured participation	1	The degree of identity of each individual
		2	The level of self-actualization of each person
		3	Preparation suitable for people
		4	Providing personal and organizational information and feedback
		5	Shared decision-making
6	The capacity of local organizations (Supporting insured)	1	Communicating with decision-makers
		2	Forming a supportive coalition
		3	Identification of root barriers
		4	Lobbying with relevant actors
		5	Monitoring and supervision the implementation and effectiveness of laws

**Table 2:**
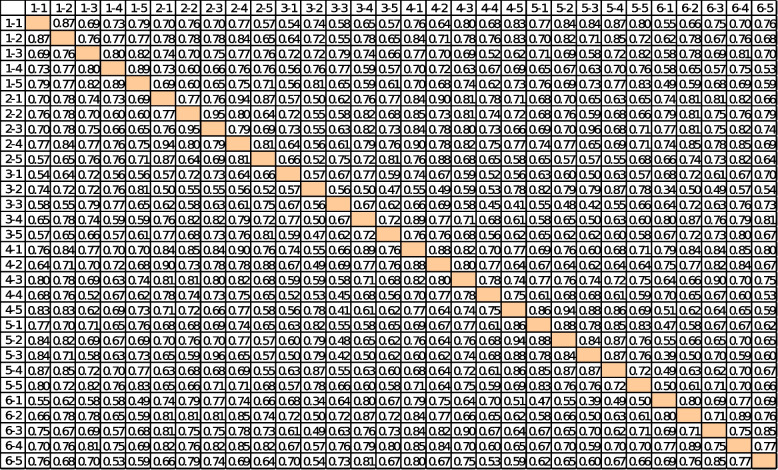
Strength of relationships matrix (SRM)

**Table 3:**
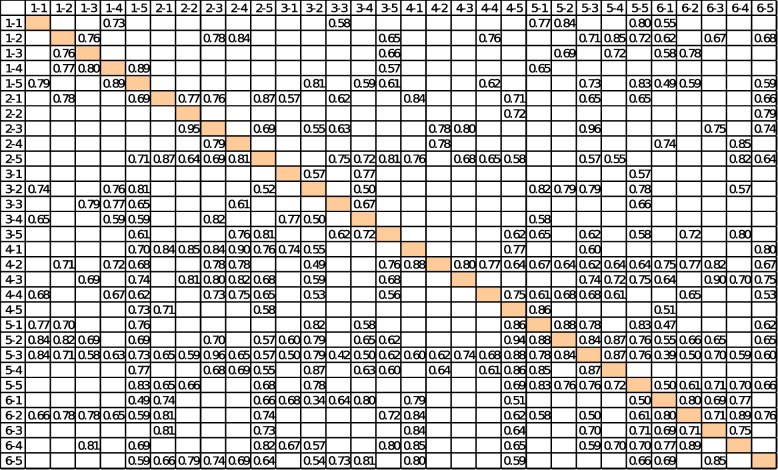
Final matrix

**Table 4:** Ranking of cognitive map variables based on centrality index

Strategy	Code	Component	Outdegree	Indegree	Centrality	Rank
Information and education (Informing the insured)	1-1	Individual factors	4.26	5.96	10.23	25
	1-2	Communication factors	8.03	6.02	14.06	19
	1-3	Spatial factors	4.19	5.89	10.08	26
	1-4	Cultural factors	3.67	6.4	10.07	27
	1-5	Social factors	7.54	14.55	22.09	4
Accountability of insured service providers	2-1	Legal and policy factors	8.56	6.74	15.3	18
	2-2	Punitive factors	1.51	6.06	7.57	29
	2-3	Control factors	6.85	10.07	16.92	13
	2-4	Motivational factors (incentives)	3.16	8.3	11.46	24
	2-5	Organizational factors	11.25	11.52	22.77	3
Financial protection of the insured	3-1	Health savings accounts	1.91	4.53	6.44	30
	3-2	Condition-based cash exchanges	7.08	10.09	17.17	10
	3-3	Supply-side subsidies	4.15	4.99	9.14	28
	3-4	Demand-side subsidies	4.5	7.94	12.44	23
	3-5	Charity attraction in the field of health	7.51	8.67	16.18	14
Protection of insured' rights (advocacy)	4-1	Drafting the rules	8.35	7.2	15.55	16
	4-2	Hospital social workers	14.23	2.82	17.05	11
	4-3	Hospital supervisory experts	11.01	3.02	14.03	20
	4-4	Virtual social networks	9.7	4.09	13.78	22
	4-5	Convince policymakers to legislate	3.39	12.03	15.42	17
Insured participation	5-1	The degree of identity of each individual	8.07	9.53	17.6	9
	5-1	The level of self-actualization of each person	13.76	6.12	19.87	6
	5-3	Preparation suitable for people	19.29	12.72	32.01	1
	5-4	Providing personal and organizational information and feedback	8.62	7.25	15.86	15
	5-5	Shared decision making	10.54	12.51	23.05	2
The capacity of local organizations (Supporting insured)	6-1	Communicating with decision-makers	8.41	9.74	18.14	8
	6-2	Forming a supportive coalition	12.05	7.68	19.73	7
	6-3	Identification of root barriers	6.58	7.45	14.03	21
	6-4	Lobbying with relevant actors	9.51	7.44	16.95	12
	6-5	Monitoring and supervision the implementation and effectiveness of laws	9.78	10.14	19.93	5

**Table 5:** The highest and lowest Outdegree and Indegree components in insured

Strategy	highest/lowest	Outdegree	Indegree	Centrality
Information and education (Informing the insured)	highest	Communication factors	Social factors	Social factors
	lowest	Cultural factors	Spatial factors	Cultural factors
Accountability of insured service providers	highest	Organizational factors	Organizational factors	Organizational factors
	lowest	Punitive factors	Punitive factors	Punitive factors
Financial protection of the insured	highest	Charity attraction in the field of health	Condition-based Cash Exchanges	Condition-based Cash Exchanges
	lowest	Health Savings Accounts	Health Savings Accounts	Health Savings Accounts
Protection of insured' rights	highest	Hospital Social Workers	Convince policymakers to legislate	Hospital Social Workers
	lowest	Convince policymakers to legislate	Hospital Social Workers	Virtual Social Networks
Insured participation	highest	Preparation Suitable for people	Preparation Suitable for people	Preparation Suitable for people
	lowest	The degree of identity of each individual	The level of self-actualization of each person	Providing personal and organizational information and feedback
The capacity of local organizations (Supporting insured)	highest	Forming a supportive coalition	Monitoring and supervision the implementation and effectiveness of laws	Monitoring and supervision the implementation and effectiveness of laws
	lowest	Identification of root barriers	Lobbying with relevant actors	Identification of root barriers

Once the necessary framework is established for people to participate in the health system, it can lead to shared decision-making between the individual and the health system and can lead to increased satisfaction with the health system as a whole. In the strategy of the capacity of local organizations and NGOs, the component with a 19.93 score (Monitoring and supervision the implementation and effectiveness of laws) has the highest centrality index. This component highlights the role of NGOs and local groups in empowering individuals. These groups, with the role of controlling and overseeing the proper implementation of laws, can have an appropriate platform for quality service delivery by healthcare providers, such as physicians. Local organizations, by exercising their supervisory and control roles, can make other people and groups more sensitive to quality in health-related services and, in addition to promoting quality in health and health insurance, can also empower individuals.

## Discussion

The method proposed in this paper is based on distance and relies on verbal variables, expert opinion and the application of a Z-number approach. This method is applicable to problems where there are qualitative data and modeling using expert information. The proposed method in this paper can serve as a decision support method for the final decision maker. The ZCM method used in modeling the empowerment of individuals in the health system has the following benefits:

- Implementation of expert knowledge in the health system.
- Using verbal variables to extract implicit knowledge of health system experts.
- Using a Z-number approach to cover ambiguity and uncertainty in the information and knowledge of health system experts. Applying this approach will modify the amount of initial comments (for example, Table 6 shows the difference in results between two states using and not using a Z-number approach an expert commenting on component 1).

**Table 6:** One expert comment on component 1

Linguistic variable	Without Z-number	With Z-number ($\widetilde{A},\widetilde{R}$)	
		$\widetilde{A}$	$\widetilde{R}$
	High	High	Medium
Triangular fuzzy number	(0.5, 0.75,1)	(0.5, 0.75,1)	(0.25,0.5,0.75)
Value	0.75	0.375

- Ability to use the model in environments with statistical data deficiencies and the existence of qualitative data with a degree of ambiguity and uncertainty (such as the issue of empowering the insured in the health system).
- Ease of use for nontechnical users.

Considering the importance and status of health and considering it as one of the development criteria for developing countries, it is important to plan and make policy based on people as the recipients of health services. Therefore, for planning and policymaking in this field, identifying effective strategies and variables affecting the empowerment of people in the health system and health insurance and identifying the cause and effect relationships between them can be very important. The cause-and-effect model of the factors affecting empowerment is thus identified by the Z-number cognitive mapping method. As seen from the model results, the strategies, insurer participation, capacity of local organizations and nongovernmental organizations (NGOs), insurer rights protection, and accountability of insured service providers have the highest scores in the centrality index (total Outdegree and Indegree), respectively. Among the components related to the strategies, the appropriate support component was found to be the most central component, with a score of 32.01, which was significantly higher than the rest of the components. It can be concluded that the paradigm shift from physician-centered to patient/person-centered in the field of health can provide the basis for empowerment. It should be noted that hospitalization for individuals should not interfere with the duties of physicians and treatment protocols but should be considered as a supplement.

In general, it can be said that the Iranian Health Insurance Organization can take into account the results and provide more insurance for its insured. An organization can use the capacity of NGOs and consult with decision-making and law-making authorities to develop and enforce laws to protect the rights of its core customers, who are the insured. The organization can outsource its activities to a purely insurer organization and define new social roles for itself. The Iranian Health Insurance Organization can play an active role by relying on its insured, who make up more than half of Iran's population, by properly implementing an empowerment-based approach and prioritizing the implementation of operational plans in accordance with specified strategies. By implementing an empowerment approach at the community level, in a given time horizon, one can see its many benefits.

Understanding how interactions between insured individuals/individual empowerment can help health system planners make better decisions is illustrated in Fig. 2. Overall, it can be concluded that empowering people can be useful as one of the effective strategies in the health system of the country and their health insurance. It can also help reduce health and insurance costs in the long run, in addition to highlighting the role of people in all matters related to their health. Applying the concept of empowerment in the field of health has made the health system more efficient and can be a clear signal for redefining the role of actors in the health system. Experience has shown that the current parametric reforms and donations, without full recognition and foresight, are unlikely to avert future crises, and changing the health system from its current disease-centered approach to an individual-centered procedure is one of the most effective ways to do so. Among the missing links in this paradigm shift is the empowerment of individuals. Health policymaking is a systematic and multifaceted process that needs to be taken into account in all aspects of its formulation. The impact of implementing each strategy on other policies must be determined before making plans operational. Using the results of this study, an empowerment approach can be implemented in the health system.

The subject of the study conducted in this article holds significant practical implications in the field of medical insurance, potentially leading to strategic changes in basic insurance organizations. Achieving success in advancing the approach of empowering individuals in the health system, a concept already recognized by the World Health Organization, can serve as a foundation for improving overall population health. Based on the findings of this research, the Iran Health Insurance Organization can consider and implement various operational programs. Taking into account the scores assigned to each component of empowering individuals in the health system can aid in prioritizing, selecting, and implementing related programs. Potential future operational plans derived from this study include designing and implementing a call center focused on increasing health awareness and providing information, establishing a system to access individuals' specific treatment and disease data, developing guidelines to prevent non-communicable diseases, fostering collaboration with non-governmental organizations and NGOs associated with common diseases such as multiple sclerosis, kidney transplantation, and various types of cancers, delivering diverse trainings on lifestyle changes, and informing the public about governmental support and treatment insurance related to their medical conditions. These operational plans, derived from the findings of this study, have the potential to drive substantial improvements in the health system and contribute to the overall well-being of the population.

One limitation of the present research pertains to the inherent challenge of conducting semi-structured and time-consuming interviews with experts. Given the qualitative nature of the employed method and the modeling process based on data extracted from Iranian experts, it is important to note that the findings of this study may not be fully generalizable to all societies and countries. However, the ranking obtained among the components of empowering individuals in the health system, as well as the reliability of the study's findings, provide a valuable foundation for conducting similar research by other scholars in different countries.

## Conclusion

This article presents an examination of the effective components that contribute to empowering individuals in the health system. Using the soft operations research approach, the study introduces and models these components, and explores the cause-and-effect relationships among them. The findings offer valuable insights for policy makers, providing a clearer understanding of the factors that drive empowerment within the health system. The identified causal relationships aid executives in decision-making and selecting effective programs. The research contributes to healthcare management and policy development, serving as a foundation for future studies. By leveraging this knowledge, we can work towards a more empowered and inclusive health system, benefiting individuals and societies at large.

### Supplementary Information

**Supplementary Material 1.**

## Data Availability

No datasets were generated or analysed during the current study.

## Abbreviations

WHO: World Health Organization
NGO: Non-governmental organization
FCM: Fuzzy Cognitive Map
SRM: Strength of Relationships Matrix
ZCM: Z-number Cognitive Map

## Indices

C_i_: Nod, concept, variable or component *i*
e_ij_: The value of the relations between C_i_ and C_j_
V_i_: Experts comment on *i* variable
S_ij_: Cause‒effect relation between the concepts *i *and *j*
X_1_(*V*_j_): The degrees of membership of in vectors *V*_1_ and *V*_2_
d*j*: The distance between the elements of j, in the vectors and *V*_1_ and *V*_2_
AD: The mean distance between vectors *V*_1_ and *V*_2_
S: similarity between vectors *V*_1_ and *V*_2_
