# Supplementary material for: Empowerment of individuals in Iranian health systems: a qualitative study using the Z-cognitive map approach
Source: BMC Health Serv Res. 2024 Apr 3;24:414. doi: 10.1186/s12913-024-10866-8 (PMC10988921; doi:10.1186/s12913-024-10866-8)
Supplement: Supplementary file 1 — Supplementary Material 1. [file 12913_2024_10866_MOESM1_ESM.docx]

Supplementary file 1

Questionnaire for statistics of experts' opinions. Used in the perceptual mapping method of Z- number based on distance

| strategy | component | Questions | Very high | high | moderate | low | very low |
| --- | --- | --- | --- | --- | --- | --- | --- |
| Information and education  (Informing the insured) | Individual factors | In your opinion, how effective is this factor in empowering the insured? |  |  |  |  |  |
|  |  | How confident are you about the answer provided? |  |  |  |  |  |
|  | Communication factors | In your opinion, how effective is this factor in empowering the insured? |  |  |  |  |  |
|  |  | How confident are you about the answer provided? |  |  |  |  |  |
|  | Local factors | In your opinion, how effective is this factor in empowering the insured? |  |  |  |  |  |
|  |  | How confident are you about the answer provided? |  |  |  |  |  |
|  | Cultural factors | In your opinion, how effective is this factor in empowering the insured? |  |  |  |  |  |
|  |  | How confident are you about the answer provided? |  |  |  |  |  |
|  | Social factors | In your opinion, how effective is this factor in empowering the insured? |  |  |  |  |  |
|  |  | How confident are you about the answer provided? |  |  |  |  |  |
| Accountability of insured service providers | Legal factors and policy making | In your opinion, how effective is this factor in empowering the insured? |  |  |  |  |  |
|  |  | How confident are you about the answer provided? |  |  |  |  |  |
|  | Punitive agents | In your opinion, how effective is this factor in empowering the insured? |  |  |  |  |  |
|  |  | How confident are you about the answer provided? |  |  |  |  |  |
|  | Controlling factors | In your opinion, how effective is this factor in empowering the insured? |  |  |  |  |  |
|  |  | How confident are you about the answer provided? |  |  |  |  |  |
|  | Motivational factors (incentives) | In your opinion, how effective is this factor in empowering the insured? |  |  |  |  |  |
|  |  | How confident are you about the answer provided? |  |  |  |  |  |
|  | Organizational factors | In your opinion, how effective is this factor in empowering the insured? |  |  |  |  |  |
|  |  | How confident are you about the answer provided? |  |  |  |  |  |
| strategy | component | Questions | Very high | high | moderate | low | very low |
| Financial protection of the insured | Health savings accounts | In your opinion, how effective is this factor in empowering the insured? |  |  |  |  |  |
|  |  | How confident are you about the answer provided? |  |  |  |  |  |
|  | Cash transactions based on conditions | In your opinion, how effective is this factor in empowering the insured? |  |  |  |  |  |
|  |  | How confident are you about the answer provided? |  |  |  |  |  |
|  | Supply side subsidies | In your opinion, how effective is this factor in empowering the insured? |  |  |  |  |  |
|  |  | How confident are you about the answer provided? |  |  |  |  |  |
|  | Demand side subsidies | In your opinion, how effective is this factor in empowering the insured? |  |  |  |  |  |
|  |  | How confident are you about the answer provided? |  |  |  |  |  |
|  | Attract good health | In your opinion, how effective is this factor in empowering the insured? |  |  |  |  |  |
|  |  | How confident are you about the answer provided? |  |  |  |  |  |
| Protection of insured rights  (advocacy) | Drafting the rules | In your opinion, how effective is this factor in empowering the insured? |  |  |  |  |  |
|  |  | How confident are you about the answer provided? |  |  |  |  |  |
|  | Hospital social workers | In your opinion, how effective is this factor in empowering the insured? |  |  |  |  |  |
|  |  | How confident are you about the answer provided? |  |  |  |  |  |
|  | Hospital supervisory experts | In your opinion, how effective is this factor in empowering the insured? |  |  |  |  |  |
|  |  | How confident are you about the answer provided? |  |  |  |  |  |
|  | Virtual social networks | In your opinion, how effective is this factor in empowering the insured? |  |  |  |  |  |
|  |  | How confident are you about the answer provided? |  |  |  |  |  |
|  | Convince policymakers to legislate | In your opinion, how effective is this factor in empowering the insured? |  |  |  |  |  |
|  |  | How confident are you about the answer provided? |  |  |  |  |  |
| strategy | component | Questions | Very high | high | moderate | low | very low |
| Insured participation | The degree of identity of each individual | In your opinion, how effective is this factor in empowering the insured? |  |  |  |  |  |
|  |  | How confident are you about the answer provided? |  |  |  |  |  |
|  | The level of self-actualization of each person | In your opinion, how effective is this factor in empowering the insured? |  |  |  |  |  |
|  |  | How confident are you about the answer provided? |  |  |  |  |  |
|  | Preparation suitable for people | In your opinion, how effective is this factor in empowering the insured? |  |  |  |  |  |
|  |  | How confident are you about the answer provided? |  |  |  |  |  |
|  | Providing personal and organizational information and feedback | In your opinion, how effective is this factor in empowering the insured? |  |  |  |  |  |
|  |  | How confident are you about the answer provided? |  |  |  |  |  |
|  | Shared decision-making | In your opinion, how effective is this factor in empowering the insured? |  |  |  |  |  |
|  |  | How confident are you about the answer provided? |  |  |  |  |  |
| The capacity of local organizations  (Supporting insured) | Communicating with decision-makers | In your opinion, how effective is this factor in empowering the insured? |  |  |  |  |  |
|  |  | How confident are you about the answer provided? |  |  |  |  |  |
|  | Forming a supportive coalition | In your opinion, how effective is this factor in empowering the insured? |  |  |  |  |  |
|  |  | How confident are you about the answer provided? |  |  |  |  |  |
|  | Identification of root barriers | In your opinion, how effective is this factor in empowering the insured? |  |  |  |  |  |
|  |  | How confident are you about the answer provided? |  |  |  |  |  |
|  | Lobbying with relevant actors | In your opinion, how effective is this factor in empowering the insured? |  |  |  |  |  |
|  |  | How confident are you about the answer provided? |  |  |  |  |  |
|  | Monitoring and supervision the implementation and effectiveness of laws | In your opinion, how effective is this factor in empowering the insured? |  |  |  |  |  |
|  |  | How confident are you about the answer provided? |  |  |  |  |  |

Supplementary file 2

Profile of experts participating in this research

| Field of activity | Years of work experience | Educational certificate | Gender | Expert number |
| --- | --- | --- | --- | --- |
| Medical center management, Management in health insurance, Hospital expert | 13 years | (G.P) & (MPH) | Man | 1 |
| Medical center management, Management in health insurance, Research in the field of public health, Medical insurance expert | 14 years | PhD in health policy & (MPH) | Woman | 2 |
| Medical center management, Management in health insurance, Research in the field of public health, Hospital expert | 18 years | PhD in health services management | Man | 3 |
| Management in health insurance, Research in the field of public health, Medical insurance expert | 11 years | PhD health services management | Man | 4 |
| Medical center management, Management in health insurance | 14 years | PhD in health policy & (MPH) | Woman | 5 |
| Management in health insurance, Research in the field of public health, Hospital expert | 18 years | (G.P) & (MPH) | Woman | 6 |
| Management in health insurance, Research in the field of public health | 13 years | PhD in health economics & (MPH) | Man | 7 |
| Management in health insurance, Hospital expert, Research in the field of public health | 13 years | PhD in health economics & (MPH) | Woman | 8 |
| Medical center management, Medical insurance expert, Hospital expert | 11 years | (G.P) & (MPH) | Man | 9 |
| Management in health insurance, Research in the field of public health | 21 years | PhD in health policy & (MPH) | Man | 10 |
| Management in health insurance,  Research in the field of public health, Medical insurance expert | 19 years | PhD in health policy & (MPH) | Man | 11 |
| Management in health insurance, Research in the field of public health, Medical insurance expert | 17 years | PhD health services management | Woman | 12 |
